# Villous Papilloma of the Rectum

**Published:** 1913-03-29

**Authors:** 


### VILLOUS PAPILLOMA OF THE RECTUM.

Benign tumours of the rectum are so uncommon in adults that they acquire very considerable importance from the fact that they are so likely to be confounded with cancer, which probably accounts for 95 per cent. or more of all rectal tumours after puberty.

The so-called villous papilloma of the rectum is, perhaps, more often mistaken for cancer than any other simple growth arising in this situation, for it often attains very considerable size, and then certainly has some points of resemblance to that form of cancer characterised by great overgrowth of soft colloid-like material, which may render exact delimitation of its extent most difficult.

The clinical history of a case of villous papilloma is usually a long one, and the two chief symptoms are hæmorrhage and discharge. But careful questioning will elicit the fact that the hæmorrhage, although profuse, only occurs when the tumour prolapses, which it generally does quite early in its career—the prolapse taking place with each action of the bowels. The discharge—unlike the dark evil-smelling discharge of cancer—is odourless and has a consistency like white of egg, and is discharged in quantities of half an ounce or more at intervals of about five or six hours.

Such a history is almost pathognomonic of the disease, but examination of the rectum is equally typical. Generally within easy reach of the finger will be discovered a soft velvety mass which has the consistency of blood-clot. At first it is difficult to make out the boundaries or attachments of the tumour, but a little persistence will gradually elicit the fact that it is attached to the wall of the rectum and that the attachment is of a linear nature, the tumour growing like a hedge, as it were, from the mucous membrane. The direction of this "hedge" of growth is always parallel or nearly parallel with the axis of the bowel, and it may be situated either anteriorly or posteriorly, and sometimes laterally. In exceptional cases the growths are multiple.

If a sigmoidoscope be now introduced, and this must be done carefully in order to avoid obscuring the field of view by blood, a very beautiful demonstration of the nature and extent of the tumour is obtained. It will be found to be two or three inches in length with a bushy top covered with small papilliferous projections, and attached to the mucous membrane throughout its length by a thin pedicle. The benign nature of the growth is proved by its free mobility and by the absence of any fixation or infiltration of the rectal wall.

Treatment of such cases is not easy and has often to be carried out in two or more stages. The patient should be placed either in the lithotomy position or on the right side and anæsthesia should be induced to the full extent. The external sphincter has probably been already overstretched by the constant prolapse of the tumour, and it is therefore wise to divide it with a scalpel rather than stretch it any further. When this has been done the growth may spontaneously prolapse, but in any case it is easy to drag it down after seizing it with pile forceps. If the tumour is a small one it will be easy to excise it and bring the edges of the mucous membrane together.

But in large tumours this is not always possible, and the pedicle must be transfixed and tied off with thick silk. Care must be taken that the needle is not passed too deeply, for if it penetrates the whole thickness of the rectal wall and enters the pouch of Douglas, pelvic peritonitis is by no means an imaginary risk.

After this has been done the main mass of the tumour is cut away and the remaining portions included in the ligatures slough off during the next nine or ten days, leaving a small scar, which in no way diminishes the lumen of the rectum.

